# Differential sub-nuclear distribution of hypoxia-inducible factors (HIF)-1 and -2 alpha impacts on their stability and mobility

**DOI:** 10.1098/rsob.160195

**Published:** 2016-09-21

**Authors:** S. E. Taylor, J. Bagnall, D. Mason, R. Levy, D. G. Fernig, V. See

**Affiliations:** Department of Biochemistry, Centre for Cell Imaging, University of Liverpool, Institute of Integrated Biology, Liverpool L69 7ZB, UK

**Keywords:** hypoxia, hypoxia-inducible factor, HIF-2α, nuclear speckles, confocal microscopy, fluorescence recovery after photo-bleaching

## Abstract

Cellular adaptation to hypoxia occurs via a complex programme of gene expression mediated by the hypoxia-inducible factor (HIF). The oxygen labile alpha subunits, HIF-1α/-2α, form a heterodimeric transcription factor with HIF-1β and modulate gene expression. HIF-1α and HIF-2α possess similar domain structure and bind to the same consensus sequence. However, they have different oxygen-dependent stability and activate distinct genes. To better understand these differences, we used fluorescent microscopy to determine precise localization and dynamics. We observed a homogeneous distribution of HIF-1α in the nucleus, while HIF-2α localized into speckles. We demonstrated that the number, size and mobility of HIF-2α speckles were independent of cellular oxygenation and that HIF-2α molecules were capable of exchanging between the speckles and nucleoplasm in an oxygen-independent manner. The concentration of HIF-2α into speckles may explain its increased stability compared with HIF-1α and its slower mobility may offer a mechanism for gene specificity.

## 1. Introduction

When cells experience oxygen deprivation, the highly conserved canonical hypoxia signalling pathway is activated. This triggers the transcription of a variety of genes supporting cell survival and restoring oxygen homeostasis, mediated by hypoxia-inducible factor (HIF). HIF is a heterodimeric transcription factor composed of an oxygen labile alpha subunit (HIF-1α or HIF-2α) and a constitutively expressed beta unit (HIF-1β, also known as ARNT).

HIF-1α was discovered in 1992 [1]. This was shortly followed by HIF-2α, which was sequenced simultaneously by different laboratories, hence published under several names: endothelial PAS domain protein-1 (EPAS1), HIF-1α-like factor (HLF) or members of PAS superfamily 2 (MOP2) [2–5]. This beta-helix–loop–helix (bHLH) protein is similar to HIF-1α in that it can form a heterodimer with HIF-1β, recognize the same DNA consensus sequence (Hypoxia Response Element/HRE) and activate transcription of hypoxia-inducible genes [3]. The expression level and transcriptional activity of HIF-2α has been shown to be regulated by the same oxygen-dependent mechanisms as HIF-1α (i.e. via the hydroxylation of specific prolyl and asparagyl residues [6,7]).

However, there are important differences between the two isoforms. First, HIF-1α and HIF-2α are encoded by different genes (*HIF1A* and *EPAS1*, respectively) and translate into proteins of different lengths (826 and 870 amino acids, respectively). These two proteins do share the same domain organization, with the highest similarity within the bHLH, PER/ARNT/SIM (PAS) and transactivation (TAD) domains, but only have an overall sequence homology of 48% [8–11]. In addition to this, HIF-2α has a unique water-filled pocket within the PAS-B domain, the purpose of which is currently unknown [12]. Most importantly, the alpha subunits are differentially regulated at all levels (transcription, translation, protein stability and protein activity), they have unique interaction partners and undergo distinct post-translational modifications (reviewed by Keith *et al.* [13]). Moreover, while the promoters of hypoxia-inducible genes have the potential to be bound by both isoforms, some are uniquely targeted by either HIF-1α or HIF-2α [14–16], and how this gene specificity arises is not currently understood.

Here, we report nuclear distribution as an additional difference between HIF-1α and HIF-2α. There are many examples of intracellular location regulating physiological processes, especially in the nucleus, where the sub-nuclear organization is integral in regulating key steps in gene expression [17,18]. The nucleus contains a rich assortment of non-membrane bound punctate structures (for example PML bodies, cajal bodies, transcription factories, as well as the nucleolus), which act as sites of protein activity, modification, complex assembly or storage [19–23]. We hypothesized that distinct sub-nuclear localization of the alpha subunits contributes to the differences in the regulation and activity of these two transcription factors.

We observed that HIF-2α exhibits a non-homogeneous, speckle-like localization within the nucleus. We showed that the number, size and motility of the HIF-2α nuclear speckles are not dependent on the level of oxygenation. We further demonstrated that while the HIF-2α molecules are moving freely in and out of the speckles, their mobility is considerably slower to the one of HIF-1α. This difference in mobility may underpin the observed differential target-gene regulation.

## 2. Results

### 2.1. HIF-2α exhibits a non-homogeneous nuclear localization

**Figure 1.**
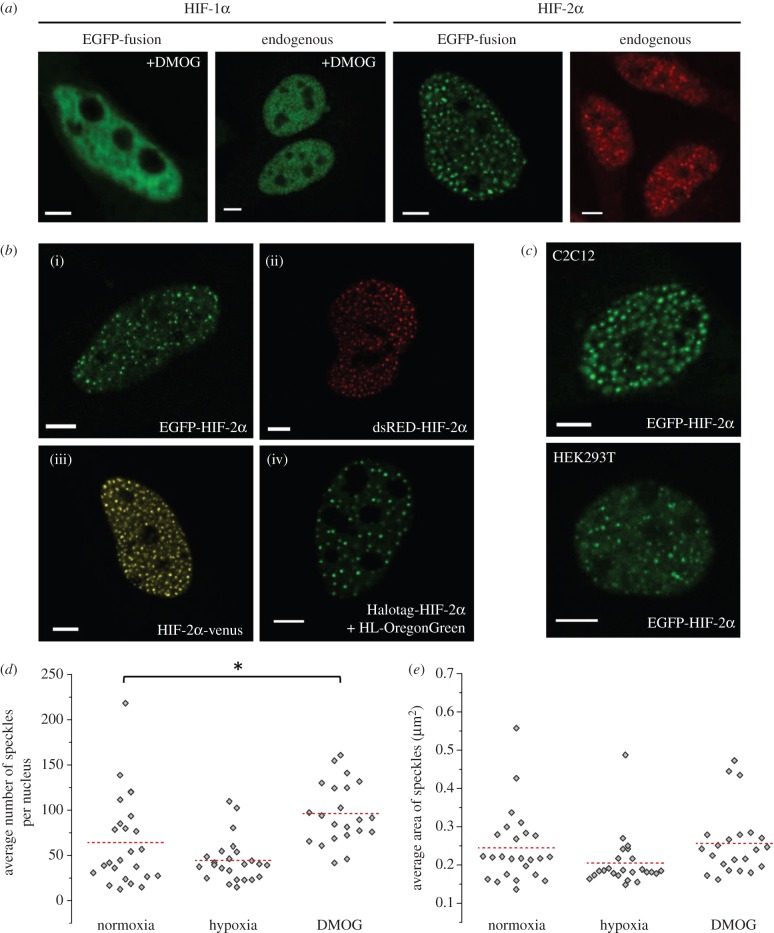
Sub-nuclear localization of HIF-1α and HIF-2α. (*a*) HeLa cells ectopically expressing HIF-1α and HIF-2α EGFP fusions compared with endogenous HIF-1α and HIF-2α labelled using immunostaining. Images of HIF-1α were taken following DMOG treatment (6 h; 0.5 mM). Scale bar, 5 μm. (*b*) HeLa cells transiently transfected with plasmids encoding (i) clover-HIF-2α (green, pseudocolour), (ii) dsRED-HIF-2α (red, pseudocolour), (iii) HIF-2α-venus (yellow pseudocolour) and (iv) Halotag-HIF-2α (green, pseudocolour). The cells expressing Halotag-HIF-2α were labelled with the fluorescent Oregon Green Halotag ligand (HL-OregonGreen; Promega, WI, USA) to visualize the fusion protein. (*c*) Confocal images of C2C12 (mouse myoblast; top) and HEK293T (Human embryonic kidney cells; bottom) cells ectopically expressing EGFP-HIF-2α. Scale bar, 5 μm. (*d*) HeLa cells transiently transfected with EGFP-HIF-2α were imaged with a CCD camera. One thousand frames were acquired per cell in normoxia, hypoxia (1% v/v O_2_, 16 h) or following treatment with DMOG (0.5 mM, 6 h). The average (±s.d.) number of speckles per nucleus in each condition was 64 ± 49 (*n* = 25), 44 ± 24 (*n* = 24) and 96 ± 33 (*n* = 22), respectively. Mean of the sample data represented by the red dashed line. (*e*) Using the images from (*d*) the average speckle area per nucleus over the 1000 frames. The mean values (±s.d.) for each condition were 0.24 ± 0.09 µm (*n* = 25), 0.21 ± 0.07 µm (*n* = 24) and 0.27 ± 0.09 µm (*n* = 22), respectively. The mean values for hypoxia and DMOG were compared with the normoxic values (independent *t*-test, significance value set at 5%). Mean of the sample data represented by the red dashed line.

We have previously investigated the temporal dynamics of both HIF-1α and HIF-2α at the single-cell level and demonstrated that they both displayed pulsatile dynamics [24]. While providing highly detailed temporal data, the time-lapse imaging did not provide images with sub-nuclear resolution; hence, the differing localization of the two HIF subunits was overlooked. Higher-resolution imaging revealed that the two alpha subunits, ectopically expressed in HeLa cells, exhibit different nuclear distribution (figure 1*a*). HIF-1α accumulates in the nucleus in a homogeneous manner, except its exclusion from the nucleolus, upon exposure to hypoxia or treatment with a hypoxia mimetic drug (DMOG). By contrast, HIF-2α localizes in punctate nuclear foci, also with nucleolar exclusion. To ensure that this observation was not due to either overexpression artefacts or GFP fusion to the HIF protein, we imaged the endogenous HIF proteins in HeLa cells by immunofluorescent staining and observed the same distributions (figure 1*a*). Moreover, the heterogeneous localization of HIF-2α fusion proteins was observed regardless of the orientation (N- or C-terminal of HIF-2α) or type of tag (e.g. fluorescent protein or Halotag; figure 1*b*). The non-homogeneous HIF-2α distribution was not unique to HeLa cells and was observed in a range of mammalian cells, including human embryonic kidney and mouse myoblast cells (figure 1*c*). These results demonstrate that the observed speckle distribution of HIF-2α is not due to artefacts of ectopic expression, presence of a tag or cell type.

To explore if this localization plays a role in the response to hypoxia, we performed quantitative analysis of images obtained from HeLa cells transiently transfected with EGFP-HIF-2α and incubated in normoxia (21% O_2_), hypoxia (1% O_2_, 8 h), or treated with DMOG to mimic the effects of hypoxia. In normoxia, we recorded 10–200 speckles per nucleus (average 64, *n* = 25) that were 0.24 (±0.07 s.d.) μm in size and take up approximately 3% of the nucleus area (figure 1*d*,*e*). The size of the speckles did not change in either hypoxia or DMOG compared with normoxia (figure 1*e*). While the number of speckles did not increase, time-lapse imaging showed that some of the existing speckles became more intense over time in hypoxia (electronic supplementary material, movie S1), suggesting that the moderate increase in HIF-2α is absorbed into existing speckles. However, with DMOG, the effects were more striking, with a significant increase of the speckle number (figure 1*d*). This is probably due to the fact that this drug fully inhibits all three PHD isoforms (as well as other hydroxylases), whereas mild hypoxia only reduces PHD activities and is consistent with the exaggerated effects (e.g. dramatic increase in HIF protein levels) usually observed following DMOG treatment [25]. The large number of additional HIF-2α molecules stabilized by DMOG might exceed the capacity of the speckles and the extra HIF-2α molecules potentially localize in additional speckles, either newly formed ones or speckles that were already present, but were undetectable due to their containing low levels of HIF-2α.

**Figure 2.**
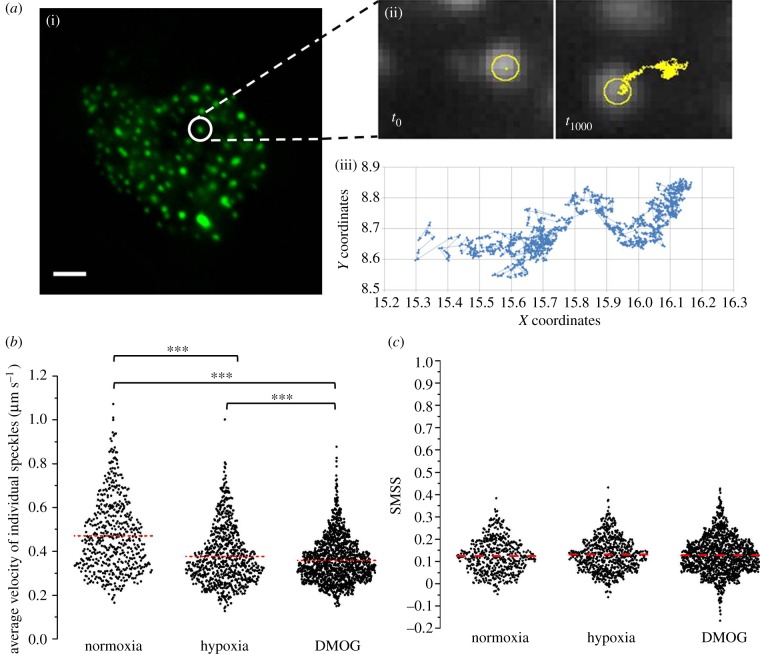
Analysis of the HIF-2α speckles trajectories. (*a*) (i) HIF-2α speckles were tracked over time. (ii) The speckles were identified in each frame and then linked frame to frame to create a trajectory map. (iii) Coordinates of these trajectories were exported to Excel and plotted. (*b*) Speed at which HIF-2α speckles move. Average (±s.d.) for normoxia 0.47 ± 0.17 µm s^−1^ (*N* = 25, *n* = 522), hypoxia 0.38 ± 0.13 µm s^−1^ (*N* = 24, *n* = 760) and DMOG 0.36 ± 0.10 µm s^−1^ (*N* = 25, *n* = 1402). Independent *t*-test, significance level 1%; normoxia compared with hypoxia: *t*_1280_ = 11.238, *p* < 0.001; normoxia compared with DMOG: *t*_1922_ = 17.954, *p* < 0.001. (*c*) SMSS values for individual speckles in normoxia, hypoxia and DMOG. Average (±s.d.) SMSS (indicated by red line) was 0.12 ± 0.07 (*N* = 25, *n* = 522), 0.13 ± 0.07 (*N* = 24, *n* = 760) and 0.12 ± 0.08 (*N* = 22, *n* = 1402), respectively.

One additional characteristic of the HIF-2α speckles, initially observed by eye through the microscope ocular, is their very fast and constrained movements within the nucleus. These movements were captured by rapid imaging (approx. 1 image per 50 ms) of single cells using a wide field epifluorescent microscope (electronic supplementary material, movie S2). Tracking the movement of the speckles revealed that the average speed was faster in normoxia compared with both hypoxia and DMOG, dropping from 0.47 µm s^−1^ (normoxia) to 0.38 µm s^−1^ (hypoxia) and 0.36 µm s^−1^ (DMOG) (figure 2*a,b*; electronic supplementary material, movies S3 and S4). To provide further insight into the motion of the HIF-2α speckles, the slope of the moment scaling spectrum (SMSS) was determined for each trajectory. This value describes the type of movement exhibited by an object. For instance, a value of 0 indicates a static object, 0.5 indicates Brownian diffusion (the movement of molecules in a suspension caused by random collisions with other molecules) and a value of 1 indicates ballistic motion (the object moves in a perfectly straight line). The intermediate types of diffusion are ‘restricted Brownian’ (same as Brownian motion, but the object's movement is restricted within an area) and ‘directed Brownian’ (diffusion is random but has overall directionality; e.g. molecular motors transporting vesicles or organelles along the cytoskeleton). Figure 2*c* shows the SMSS values of individual trajectories of speckles across the three conditions tested. The average SMSS was 0.12–0.13 with 63% of speckles analysed falling into the restricted Brownian group, while the remainder were static. There was no significant difference in the average SMSS value or the proportion of speckles within each type of diffusion category (in this case restricted Brownian or static) when compared with hypoxia and DMOG treatments. These results suggest that the speckles are physically hindered from diffusing out of a localized region. It is likely that they are trapped or, like other nuclear bodies, tethered to a physical structure (such as the nuclear scaffold) [26]. Taken together these results suggest that HIF-2α is localizing at predetermined sites with a finite size and capacity. A similar observation was previously made for HIF-1α [27].

### 2.2. HIF-2α speckles co-localize with active transcription sites

**Figure 3.**
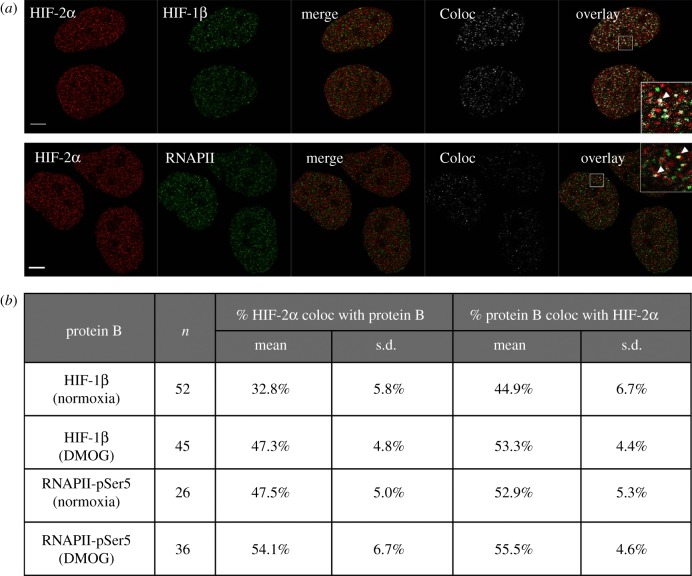
Co-localization of HIF-2α with HIF-1β and RNAPII. (*a*) Immunofluorescence detecting indicated proteins using antibodies labelled with Alexa Fluor 555 (red, pseudocolour) and Alexa Fluor 488 (green, pseudocolour). ‘Merge’ is the red image superimposed onto the green image of the co-stained nuclear proteins. ‘Coloc’ is the co-localization channel calculated using ImageJ plugin Co-localization Threshold. White indicates pixels where both red and green signal is found (i.e. co-localization). ‘Overlay’ is the co-localization image superimposed onto the merged image. Inlay is the magnified region (white square). White arrows highlight regions of co-localization. Scale bar, 5 μm. Abbreviations: RNAPII, RNA Polymerase II phospho-serine 5. (*b*) Immunofluorescence images were analysed using ImageJ plugin Co-localization Threshold with use of the Costes *et al.* [31] method to automatically create a threshold prior to calculating the Mander's coefficient for both proteins. The results are given as, for example, the percentage of protein A (HIF-2α) that co-localized with protein B (HIF-1β or RNAPII) and vice versa.

Many nuclear bodies have specific marker proteins, for example nucleolin in the nucleoli or coilin for Cajal bodies [28,29]. We used immunofluorescence and co-localization analysis to investigate the spatial relationship between HIF-2α and other nuclear proteins with a view to determining whether HIF-2α is localizing at known nuclear bodies. To provide a benchmark and validate the approach, we first measured HIF-2α co-localization with its known binding partner HIF-1β, in normoxia and upon DMOG treatment. Quantification using a Manders analysis [30] revealed that 33% of HIF-2α co-localizes with HIF-1β in normoxia which increases to 47% following treatment with DMOG (figure 3*a*,*b*; statistically significant; independent *t*-test; *p* < 0.001). As HIF-2α is a transcription factor, it was logical to investigate whether HIF-2α localizes at sites of active transcription. Immunofluorescence was performed using an antibody against phospho(ser5)–RNAPII as a marker for active transcription sites [32]. Figure 3*a*,*b* shows that 48% of HIF-2α co-localizes with the initiating form of RNAPII in normoxia, which rose to 54% in the presence of DMOG (statistically significant; independent *t*-test; *p* < 0.001). These results indicate that in each case half of the signal from HIF-2α co-localizes with its dimerization partner HIF-1β and with active transcription sites in hypoxia mimetic conditions (DMOG).

**Figure 4.**
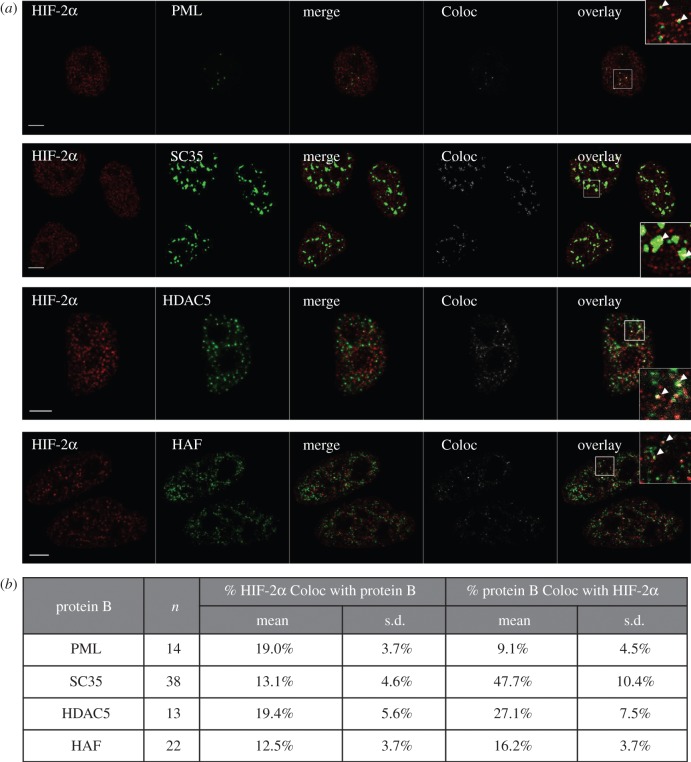
Co-localization HIF-2α and other nuclear proteins. (a) Immunofluorescent images of HIF-2α and other nuclear proteins that are known to localize into nuclear bodies. Endogenous HIF-2α is labelled with a secondary antibody coupled to Alexa Fluor 555 (red, pseudocolour). Other nuclear proteins (SC35 and HAF) were detected with a secondary antibody coupled to Alexa Fluor 488 (green, pseudocolour) or were fused to YFP for direct detection (PML and HDAC5). ‘Merge’ is HIF-2α (red) image superimposed onto the green image of the co-detected nuclear protein. ‘Coloc’ is the co-localization channel calculated using ImageJ plugin Co-localization Threshold. White indicates pixels where both red and green signal is found (i.e. co-localization). ‘Overlay’ is the merge image with the colocalization image superimposed. Inlay is the magnified region (white square). White arrows highlight regions of co-localization. Scale bar, 5 μm. Abbreviations: PML, Promyelocytic leukemia protein; HDAC5, histone deacetylase 5; HAF, hypoxia associated factor; YFP, yellow fluorescent protein. (*b*) Immunofluorescent images (*a*) were analysed using ImageJ plugin Co-localization Threshold as in figure 3.

We then investigated HIF-2α co-localization with other nuclear proteins that are known to concentrate into established nuclear bodies. Transcription factors, such as p53 and NRF2, have been shown to localize to PML bodies, suggesting that one function of PML bodies is the storage of inactive transcription factors [33,34]. Initial analysis of immunofluorescent labelling of HIF-2α in HeLa cells stably transfected with PML-YFP (figure 4*a*) indicated that HIF-2α speckles are not PML bodies as they differ in size (typically 0.5 µm for PML bodies compared with 0.24 µm for HIF-2α speckles) and number (only 10–30 PML bodies per cell) compared with HIF-2α speckles [20]. Co-localization analysis showed that only 18% of HIF-2α is co-localized with PML (figure 4*b*) suggesting that PML bodies cannot account for all inactive HIF-2α. In normoxic conditions, it is expected that a substantial proportion of HIF-2α is inactive that is not accounted for here and so may be localized at other storage sites or stalled at the promoters of target genes primed for transcriptional activation.

Other well-studied nuclear bodies are SC35 domains, also known as splicing factor compartments (SFCs) or interchromatin granule clusters (IGC), which house inactive splicing factors, such as SC35 [35,36]. Only 13% of HIF-2α co-localized with SC35 (figure 4*b*). HDAC5 is a histone deacteylase and localizes in domains that have been termed matrix associated deacetylase (MAD) bodies [37]. Images of this protein published by Downes *et al.* [37] show a sub-nuclear localization pattern very similar to that of HIF-2α, which we also observed (figure 4*a*). We found that 19% of HIF-2α co-localized with HDAC5-YFP (figure 4*b*), highlighting that there is no clear link between the two proteins even though a similar nuclear distribution pattern is exhibited. Finally, we investigated the localization of hypoxia associated factor (HAF also known as SART1_800_). This protein has been demonstrated to interact specifically with HIF-2α [38]. However, only 12% of HIF-2α was found to co-localize with HAF (figure 4*b*). Overall, none of the aforementioned nuclear bodies stand out as a clear compartment where the majority of HIF-2α concentrates. The strongest co-localization was observed with its binding partner HIF-1β and at active transcription sites. This suggests that a large proportion of the non-homogeneous localization is associated with HIF-2α function as a transcription factor.

### 2.3. Mobility of HIF-2α

**Figure 5.**
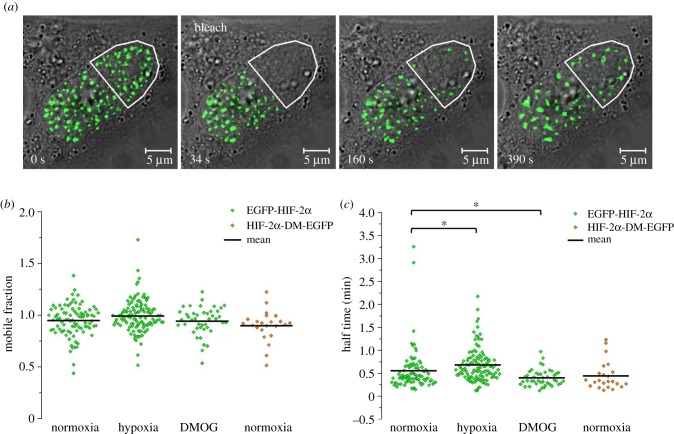
Fluorescence recovery after photo-bleaching (FRAP) of EGFP-HIF-2α. (*a*) A series of confocal images of HeLa cell ectopically expressing EGFP-HIF-2α that has been photo-bleached in half of the nucleus. Images at different time points showing gradual recovery of fluorescence into the bleached region (outlined in white). (*b*) The mobile fraction of EGFP-HIF-2α molecules per nucleus. The average (±s.d.) mobile fraction of EGFP-HIF-2α was 0.95 ± 0.15 (*n* = 89) in normoxia, 0.99 ± 0.16 (*n* = 115) in hypoxia and 0.94 ± 0.13 (*n* = 43) following treatment with DMOG. For HIF-2α-DM-EGFP, the mobile fraction was 0.9 ± 0.15 (*n* = 23). (*c*) The time taken for recovery to reach half the final intensity (*t*_half_) per nucleus in normoxia, hypoxia and DMOG. The average (±s.d.) half-time was 0.56 ± 0.46 (*n* = 89) minutes, 0.68 ± 0.36 (*n* = 115) minutes and 0.40 ± 0.18 (*n* = 43) minutes, respectively. The half-time for HIF-2α-DM-EGFP was 0.44 ± 0.31 (*n* = 23) minutes. An independent *t*-test (*α* = 0.05) was used to compare the mean normoxic half-time to those from the hypoxic (*t*_202_ = 2.122, *p* = 0.35) and hypoxia mimic (*t*_130_ = 2.156, *p* = 0.033) conditions. The mean value is represented by a black line on graph.

We next sought to determine if the localization into speckles could impact the diffusion of HIF-2α molecules and hence its availability to bind DNA promoter regions. To measure the molecular mobility of HIF-2α molecules, we utilized fluorescence recovery after photo-bleaching (FRAP). HeLa cells ectopically expressing EGFP-HIF-2α were incubated in normoxia, hypoxia (16 h pre-incubation in 1% (v/v) O_2_) or DMOG (6 h treatment; 0.5 mM). Half the nucleus of individual EGFP-HIF-2α positive cells was bleached and recovery into the bleached region was measured (figure 5*a*). For each cell, the fractional recovery over time was plotted and fitted using a one-component exponential equation. The mobile fraction (the fraction of molecules freely diffusing) and half-time (time taken for the fluorescence in the bleached region to reach half the eventual recovery) of HIF-2α were recorded from the fitted curves. The average mobile fraction was 96% (figure 5*b*), and this was not altered by incubation in hypoxia or DMOG treatment, suggesting that HIF-2α molecules are freely diffusing through the nucleus regardless of the oxygen environment. The average half-time for recovery was calculated to be 34 (±28 s.d.), 41 (±22 s.d.) and 24 (±11 s.d.) seconds in normoxia, hypoxia and DMOG conditions, respectively (figure 5*c*). Although comparison of the mean values measured for the control (normoxia) with those for hypoxia or DMOG treatment were statistically significant, the actual values were only marginally different and the distributions largely overlapped. In addition, there was no obvious trend between hypoxia and DMOG compared with the control. Therefore, the differences observed may not have a biological significance. FRAP was also performed on a constitutively stable HIF-2α mutant, where the two prolyl residues within the oxygen-dependent degradation (ODD) domain have been substituted for alanines and so cannot undergo PHD-dependent degradation. The mobile fraction and half-time for recovery were found to be very similar to that of wild-type HIF-2α, again suggesting that O_2_-dependent stabilization of HIF-2α does not affect its diffusion parameters.

**Figure 6.**
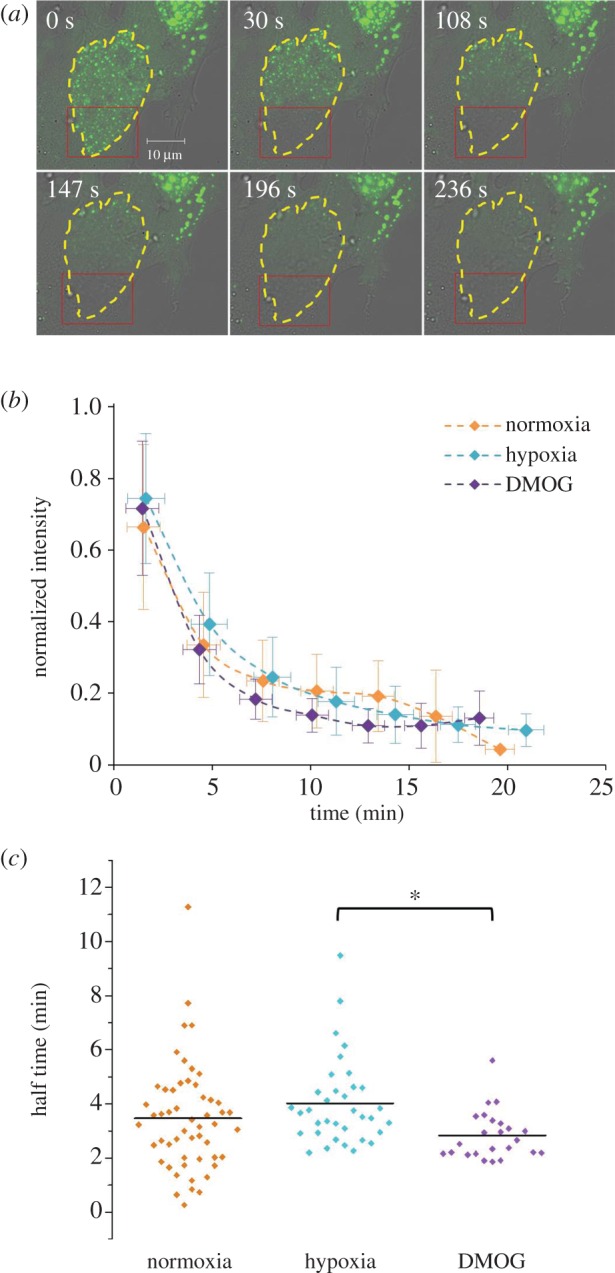
Molecular mobility of HIF-2α measured using FLIP. (*a*) Confocal images of a HeLa cell ectopically expressing EGFP-HIF-2α that has been continually bleached in one region (red box). Nucleus outlined in yellow. (*b*) The average trend of fluorescence loss in normoxia, hypoxia and DMOG. *Y*-error bars represent standard deviation. The data were grouped (‘binned’) based on time and the *X*-error bars represent the standard deviation of these bins. (*c*) Half-time values were extrapolated from the curves fitted (as in (*b*)) for each cell in the three conditions. Average half-time (represented by black line on graph) ± s.d.: normoxia = 3.47 ± 1.98 min (*n* = 53), hypoxia = 3.96 ± 1.61 min (*n* = 36), DMOG = 2.88 ± 0.88 min (*n* = 23).

To confirm the observations made, fluorescence loss in photo-bleaching (FLIP) was performed. Here, the EGFP-HIF-2α positive cells were continually bleached in the same region of the nucleus and the fluorescent signal in the non-bleached region was measured (figure 6*a*). Figure 6*b* shows the general trend of fluorescence loss in the three conditions. The fluorescence loss data were fitted using a one-component exponential equation. The resulting curve was used to determine the half-time of EGFP-HIF-2α loss of fluorescence (figure 6*c*). Overall, these results show no difference in the molecular mobility of EGFP-HIF-2α between the three conditions, supporting the FRAP data. However, this experiment highlights that HIF-2α must be continually moving in and out of the speckles (and throughout the nucleus) for all EGFP-HIF-2α to be bleached within 15 min.

We also performed some spatial analysis on the FRAP experiments by tracking the speckles during bleaching and have observed some cases of recovery in the same place (electronic supplementary material, movies S5–S7), complementing the SMSS data and the possibility that these speckles might be tethered structures in specific locations. In conclusion, our data show that HIF-2α is dynamically associated with nuclear speckles, with the HIF-2α freely diffusing in and out of them.

### 2.4. Comparing the molecular mobility of HIF-2α with HIF-1α

**Figure 7.**
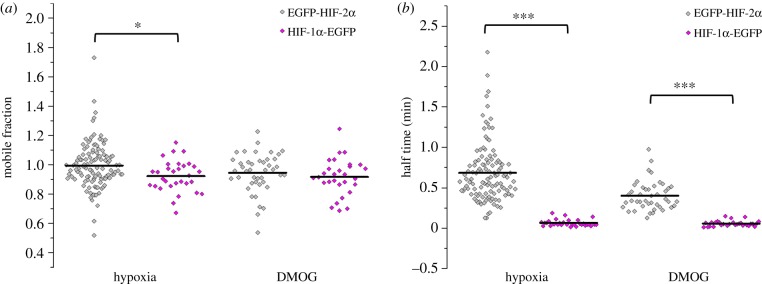
Comparing molecular availability and mobility of HIF-1α and HIF-2α. (*a*) The mobile fraction of EGFP-HIF-2α compared to HIF-1α-EGFP. The average (±s.d.) mobile fraction and half-time for EGFP-HIF-2α are the same as in figure 5. Only hypoxia and DMOG conditions are compared as HIF-1α-EGFP cannot be observed in normoxia and so cannot be photo-bleached in this condition. The average (±s.d.) mobile fraction of HIF-1α-EGFP per nucleus was 0.92 ± 0.11 (*n* = 31) hypoxia and 0.92 ± 0.12 (*n* = 29) following treatment with DMOG (0.5 mM, 6 h). Independent *t*-test (*α* = 0.05) revealed that the mobile fraction of HIF-1α-EGFP in hypoxia is significantly less than EGFP-HIF-2α (*t*_144_ = 2.412, *p* = 0.017). (*b*) The half-time recovery of EGFP-HIF-2α compared with that of HIF-1α-EGFP in hypoxia and DMOG. In both hypoxic (independent *t*-test: *t*_144_ = 9.532, *p* < 0.001) and DMOG (independent *t*-test: *t*_70_ = 10.278, *p* < 0.001) conditions the half-time of HIF-1α-EGFP was significantly less than EGFP-HIF-2α. Average values represented by black line on graph.

We compared the molecular mobility of HIF-2α with that of HIF-1α. FRAP experiments were only performed on HIF-1α-EGFP in hypoxia or following DMOG treatment, as there is no detectable fluorescent protein in normoxia, consistent with Bagnall *et al.* [24]. It was found that the mobile fraction of HIF-1α-EGFP was 92% in both conditions, which is lower than that of EGFP-HIF-2α in hypoxia (figure 7*a*). These findings could be due to the fact that FRAP looks at the population of molecules and so if sufficient HIF-1α is bound at promoters due to its role in the acute response to hypoxia, then a global effect on the mobility could be observed. However although significant, the difference between HIF-1α and HIF-2α was small (7%).

Conversely, the difference in half-time is much greater (figure 7*b*). The measurements suggest that HIF-1α-EGFP is moving more than 10-fold faster than EGFP-HIF-2α in hypoxia and 5-fold faster in the presence of DMOG. The rapid recovery of HIF-1α is typical of that seen with other transcription factors [39]. The slower recovery of mobility of HIF-2α is unusual, but could simply be explained by its concentration in nuclear speckles, which physically impedes its mobility, whereas HIF-1α-EGFP is dispersed homogeneously throughout the nucleus with only obstructions such as chromatin and molecular crowding to hinder its movement.

The speckle-like localization of HIF-2α does not seem to be affected by hypoxia and the associated change in HIF-2α transcriptional activity, but it creates a difference in terms of availability for recruitment on target genes promoters compared with HIF-1α. The dramatically different diffusion parameters and the differential local concentrations of the isoforms will probably affect availability for binding and could explain the differential promoter regulation despite them binding to the same DNA motif.

## 3. Discussion

Heterogeneous localization of HIF-2α has been previously noted by others [40] and has attracted further investigation in terms of cofactor binding [41,42]. However, the role of this localization and potential oxygen-dependent regulation has not been explored. Here, we show that the speckle localization of HIF-2α is not altered by varying oxygen levels. Instead, the speckles seem to be domains that are associated with or ‘tethered’ to a structure such as the nuclear matrix, which enables the concentration of HIF-2α in specific parts of the nucleus, particularly in close proximity to active RNA polymerase. Although HIF-2α can freely move within the entire nucleus, its slow diffusion time compared with its homologue HIF-1α and its precise localization in highly concentrated speckles point to a novel mechanism to differentiate HIF-1α and HIF-2α in their access to DNA binding regions, which may contribute to their promoter specificity.

### 3.1. Speckle-like organization of transcription factors: a mechanism for regulating activity

Hendzel *et al.* [26] proposed that there are ‘transcription factor-enriched foci’ within the nucleus that concentrate functionally related proteins to streamline assembly of macro-molecular complexes, which in turn control the concentration of the proteins in the nucleoplasm. The localization into nuclear bodies acts as an additional mechanism for controlling gene expression, as the transcription factors, whether active or not, are not able to access the promoters of target genes. The storage of active transcription factors near to active transcription sites facilitates a rapid change in gene expression, which is vital when responding to environmental stress such as oxygen deprivation.

In normoxia, we can detect both endogenous and ectopically expressed HIF-2α, in a punctate sub-nuclear distribution. Given this, we initially hypothesized that HIF-2α is regulated spatially, via an alternative or additional mechanism compared with HIF-1α (i.e. HIF-2α subunit accumulates in nuclear speckles and is physically impeded from activating genes in normoxia). However, the data obtained do not support this hypothesis, at least not directly. Photo-bleaching experiments suggest that HIF-2α is not trapped in the nuclear speckles. We also did not observe a change in the mobile fraction of HIF-2α in relation to oxygen levels, which could be expected if the speckles were storage sites controlling transcriptional activity via the availability of HIF-2α in the nucleoplasm. However, it is possible that HIF-2α is localizing at a number of sites that have different functions. For example, CREB binding protein (CBP) has been shown to localize with p300 in small foci in the nucleus [26], but also to transiently localize at PML bodies [43]. There is a possibility that there are different sub-populations of HIF-2α, some activating genes, others forming transcriptional complexes and the remainder being sequestered. This may explain the co-localization results were 20% of HIF-2α co-localized with PML or HDAC5 for example (figure 4*b*) and 50% co-localized with active transcription sites (pser5-RNAPII; figure 3*b*).

### 3.2. Differential molecular mobility of HIF-1α and HIF-2α: a possible explanation for gene specificity

It is becoming increasingly evident that the HIF-2α isoform has specific roles and unique target genes. One conundrum is how this gene specificity arises. Mole and co-workers [44] performed genome-wide analyse of HIF-1 (HIF-1α + HIF-1β) versus HIF-2 (HIF-2α + HIF-1β) binding and found no obvious difference in the consensus sequence (HRE) or proximal sequences of genes specifically targeted by each isoform concluding that the specificity does not lie within the DNA sequence. Moreover, Ratcliffe and co-workers found that when both isoforms are present certain genes will be preferentially bound by HIF-1α; however, when HIF-1α is knocked-down, HIF-2α is found to be bound at the promoters of these genes (Peter Ratcliffe 2015, personal communication). Here, we have shown that the two isoforms have different molecular mobility in the nucleus and so the preferential binding could be explained by the difference in molecular mobility and local concentration. Therefore, we propose that different nuclear diffusion times could be a mechanism for HIF-1α/HIF-2α gene specificity.

### 3.3. Dynamics in the nuclear compartment

Many of the well-studied nuclear domains such as PML bodies have been shown to be associated with the nuclear matrix and have components responsible for structural integrity [37,43]. Tracking the individual HIF-2α speckles revealed that they are highly mobile within a confined region. In addition, visual inspection of the FRAP experiments suggests that the fluorescence recovers in the same locations as pre-bleach (figure 5*a*; electronic supplementary material, movies S5–S7). Taken together, these results suggest that HIF-2α localizes at pre-existing nuclear domains that are tethered to, or spatially restricted by, a structure such as the nuclear matrix.

It has been shown that many transcription factors and other nuclear proteins are highly dynamic within the nucleus [36,39,43]. Even when active, transcription factors are continually moving or ‘searching’ for binding sites and when a target gene is located they only have a short residency time, ranging from milliseconds to tens of seconds [39,45]. Therefore, a change in mobility due to transcriptional activation would be difficult to detect using photo-bleaching techniques, which measures the average mobility of a population of molecules. Single molecule tracking could prove a more valuable method to analyse the movement of HIF-2α molecules through the nucleoplasm and allow the determination of the residency time of single HIF-2α molecules in the nuclear speckles and at sites of transcription. Nevertheless, HIF-2α clustering into distinct speckles is a highly dynamic process, which may not only contribute to its protection from degradation but also act to regulate mobility and concentration in order to fine-tune its availability and activity.

## 4. Material and methods

### 4.1. Cell culture and treatment

#### 4.1.1. Cells

HeLa (Human cervix epitheloid carcinoma, ECACC no. 93021013) cells were grown in minimum essential medium (MEM), 10% v/v fetal bovine serum (FBS) and 1% v/v non-essential amino acids (NEAA). PML-YFP HeLa cells (a generous gift from E. G. Jaffrey, University of Dundee, UK) stably express PML-YFP [46] and were maintained in MEM, 10% v/v FCS, 1% v/v NEAA, 1 µg ml^−1^ Blasticidin (Sigma Aldrich, MO, USA) and 1% penicillin/streptomycin (Invitrogen, CA, USA). HEK293T (Human embryonic kidney, #LV900A-1; System Biosciences, Inc., CA, USA) cells were maintained in Dulbecco's Modified Eagle Medium (DMEM, #41966-029) supplemented with 10% v/v FBS. C2C12 (mouse myoblast, ECACC no. 91031101) were grown in DMEM (#41966-029) supplemented with 10% FBS and 1% Pen/Step (Sigma). All cells were cultured in humidified air at 37°C in a Sanyo CO_2_ (CO_2_ set at 5%) incubator. All cell culture reagents were from Life Technologies (CA, USA) unless stated otherwise.

#### 4.1.2. Hypoxic incubation and pharmacological treatments

Incubation conditions were as follows: normoxia (20% O_2_ v/v, 5% CO_2_ v/v, 37°C; Sanyo Electric Biomedical Co., Japan); hypoxia (1% O_2_ v/v, 5% CO_2_ v/v, 37°C; Don Whitley Scientific, UK). The hypoxia mimetic dimethyloxaloglycine (DMOG) was from Enzo Life Sciences (NY, USA) and used at a final concentration of 0.5 mM.

### 4.2. Plasmids and transfection

Plasmids encoding fluorescent HIF-1α and HIF-2α fusion proteins were as described by Bagnall *et al.* [24]. The plasmid encoding constitutively active HIF-2α (pDONR-HIF-2α-DM) with mutations on both proline residues within the ODD was kindly provided by Edurne Berra (CiCBiogune, Bilbao) and was used to create pG-HIF-2α-DM-EGFP. pCMX-PL1-YFP-mHDAC5 was a generous gift from R. M. Evans (The Salk Institute, CA, USA). HaloTag-HIF-2α was purchased at Promega (WI, USA).

For live microscopy experiments, 1.5 × 10^5^ HeLa cells were plated in a 35 mm glass bottom dish (Greiner Bio-One Ltd, Stonehouse, UK) 24 h before each imaging experiment was carried out. Cells were transiently transfected 24 h prior to imaging experiment using FuGene6 (Roche, Basel, Switzerland) following the manufacturer's protocol. A total of 1 µg plasmid DNA was used per transfection in a ratio 2 : 1 (transfection reagent: DNA).

### 4.3. Time-lapse confocal microscopy

Cells expressing EGFP-HIF-2α were imaged using a LSM 780 (Zeiss, Oberkochen, Germany) confocal microscope fitted with an incubation system and O_2_controller (PeCon GmBh, Erbach, Germany) that maintains normoxic or hypoxic conditions on the microscope stage. Cells were observed using a Plan-apochromat 63 × 1.4 oil immersion objective. EGFP was excited with an Argon ion laser at 488 nm. Images were captured using Zen 2012 software (Zeiss, Oberkochen, Germany).

### 4.4. Immunofluorescence and co-localization analysis

#### 4.4.1. Immunofluorescence

Cells were seeded at a density of 1 × 10^5^ glass^−1^ cover slip, 24 h before treatment. Following treatment (hypoxia/DMOG), cells were rinsed three times with phosphate-buffered saline (PBS; Sigma Aldrich, MO, USA) and subsequently fixed for 15 min with 4% paraformaldehyde (Sigma Aldrich, MO, USA) at room temperature, followed by three 10 min PBS washes. In total, 50 mM NH_4_CL was added for 20 min, removed and the cells were blocked for 20 mins (blocking buffer: 1% BSA (Sigma Aldrich, MO, USA), 0.1% Triton X-100 (Sigma Aldrich, MO, USA) and 0.4% Tween 20 (Sigma Aldrich, MO, USA) in PBS). Cells were then incubated for 1 h with primary antibody diluted in blocking buffer at room temperature. Cells were washed three times in blocking buffer and incubated for 30 min at room temperature with the secondary antibody (diluted in blocking buffer). The following antibodies were used: rabbit anti-HIF-2α (1 : 500; #ab20654/1 : 100; #ab179825, Abcam, Cambridge, UK), mouse anti-HIF-1α (1 : 1000; #610959, BD Biosciences, CA, USA), mouse anti-HIF-1β (1 : 100; #NB100-124, Novus Biological, CO, USA), mouse anti-RNAPII phospho ser5 (1 : 50; #ab24759, Abcam, Cambridge, UK), mouse anti-SC35 (1 : 1000; #ab11826, Abcam, Cambridge, UK), mouse anti-Sart1 (10 µg ml^−1^; #ab88583, Abcam, Cambridge, UK), anti-rabbit Alexa Fluor 555 (1 : 1000; #A-21428, Invitrogen, CA, USA) and anti-mouse Alexa Fluor 488 (1 : 500, #A-11008, Invitrogen, CA, USA). Topro-3 Iodide (diluted 1 : 1000 in PBS; Invitrogen, CA, USA) was used to stain the nuclei. Samples were imaged with a Plan-Fluar 40×/NA 1.30 oil immersion objective on a LSM 710 confocal microscope (Zeiss, Oberkochen, Germany). Alexa Fluor 488 was excited with 488 nm Argon laser, Alexa Fluor 555 was excited with 561 nm HeNe1 laser and Topro-3 Iodide was excited with 633 nm laser. Images were captured using Zen 2010 software (Zeiss, Oberkochen, Germany).

#### 4.4.2. Image analysis

Post-acquisition processing was carried out using ImageJ [47]. The middle slice from each z-stack was analysed. The background was subtracted for both the red and green channel (pixel size of 5 for HIF-2α, 5 for RNAPII, 10 for HDAC5-YFP). Analysis was performed with an ImageJ plugin for co-localization analysis (http://fiji.sc/Colocalization_Threshold).

### 4.5. Epifluoresence microscopy (speckle characterization and tracking)

Cells expressing EGFP-HIF-2α were mounted on the stage of an Axio Observer Z.1 Epifluorescent microscope (Zeiss, Oberkochen, Germany) fitted with an incubation system and O_2_ controller (PeCon GmBh, Erbach, Germany). Images were acquired with an Andor iXon 879 (16 µm pixels 512 × 512) camera (Andor Technology, Belfast, UK) using a 63× objective and 2.5× optovar lens.

#### 4.5.1. Image analysis

Number and size: calibrated image stacks (1000 frames) were analysed using ImageJ [48]. The image stack was duplicated: one was processed as follows. Background was subtracted using a 5 pixel filter and a mask applied (threshold type Li, dark background). To separate touching objects a watershed was applied and any holes were filled (using Fill Holes option). The masked images was analysed (size of pixel = 10-infinity) and measurements were redirected to the original (unprocessed) image.

Dynamics: image stacks (1000 frames) were analysed with the plugin Particle Tracker 2D/3D [49] (parameters using radius = 5, cut-off = 1, per cent = 5, link = 1, displacement = 5) in ImageJ. The trajectory coordinates were exported to Excel (Microsoft Corporation, WA, USA). Analysis of trajectories of individual speckles was performed using an in-house written macro. The velocity and slope of the moment scaling spectrum (corresponds to diffusion mode) were calculated based on formulae in Ewers *et al.* [50], Sbalzarini & Koumoutsakos [49] and Schweizer [51].

### 4.6. Fluorescence recovery after photobleaching

FRAP was performed on an Axiovert 200M LSM510 (Zeiss, Oberkochen, Germany) confocal microscope. Ten pre-bleach and 290 post-bleach images were acquired every 300 ms, using a 63× oil immersion objective. Bleaching of a region of interest (ROI) was performed with an Argon ion laser (488 nm) at 100% output power for 50 iterations. The pinhole was set at approximately 3 airy units. EGFP was excited with an Argon ion laser at 488 nm and emitted light was reflected by a 540 nm dichroic mirror through a 505–550 nm bandpass filter and detected through a 530 nm longpass filter. Data were captured by LSM510 version 3 software (Zeiss, Oberkochen, Germany).

#### 4.6.1. Analysis

For each FRAP experiment, fluorescent intensities for the bleached region (ROI1), non-bleached region (acquisition control; ROI2) and background (ROI3) were extracted using the LSM510 software. ROI1 and ROI2 were background subtracted (average) and normalized to their respective pre-bleach values (average). The resulting ROI1 values were normalized to the ROI2 values (ROI1/ROI2). This ratio was then corrected for any non-specific bleaching during acquisition using the following equation:
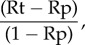
where Rt is the bleach value at a given time point and Rp is the first post-bleach value. These values were plotted against time and the curves were fitted using the following equation:
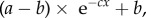
where *a* is the value of *Y* intercept, *b* the value of *Y* at infinity and *c* the rate constant for graph.

Both the normalization and curve fitting was performed in Matlab (MathWorks, MA, USA) using an in-house written code (electronic supplementary material, document 1). From the curves, the mobile fraction and half-time for each FRAP experiment was calculated.

Owing to the rapid recovery exhibited by HIF-1α-EGFP, the experimental set-up was altered slightly whereby a strip across the nucleus was bleached and was performed at 100% output power for 50 iterations.

### 4.7. Fluorescence loss in photo-bleaching

FLIP was performed on a LSM780 (Zeiss, Oberkochen, Germany) confocal microscope. Following the acquisition of 10 images, cells expressing EGFP-HIF-2α were bleached within an ROI with an Argon ion laser (488 nm) for 100 iterations at 100% output power. Twenty post-bleach images were captured before bleaching was repeated. Cycles of imaging and bleaching were carried out until fluorescence in the non-bleached region was lost. Images were captured by Zen 2012 software (Zeiss, Oberkochen, Germany).

#### 4.7.1. Analysis

For each FLIP experiment, the fluorescent intensities for the bleached region (ROI1), non-bleached region (ROI2) and background (ROI3) were extracted using Fiji. The average of the background fluorescence over the course of the time-lapse was subtracted from each ROI2 value. These background corrected values were then normalized to the average of the pre-bleach fluorescence values. These data were plotted against time. The half-time for each experiment was calculated by fitting each FLIP curves with a one component exponential decay curve in Matlab (MathWorks, MA, USA).

## Supplementary Material

Supplemental Document 1

## Data accessibility

Seven supplemental movies and the Matlab code supporting this article have been uploaded as part of the electronic supplementary material. All raw imaging data are openly available through OMERO at the following link: http://tiny.cc/2016-taylor.
