# Supplementary material for: Differential sub-nuclear distribution of hypoxia-inducible factors (HIF)-1 and -2 alpha impacts on their stability and mobility
Source: Open Biol. 2016 Sep 21;6(9):160195. doi: 10.1098/rsob.160195 (PMC5043584; doi:10.1098/rsob.160195)
Supplement: Supplemental Document 1 [file rsob160195supp1.docx]

%% FRAP ANALYSIS

% For analysis of FRAP data in excel spreadsheet format or CSV format with

% Time, Bleached Region, Non-bleached Region and Background columns.

% After running the code, the end product will be the value of Y at time infinity, the rate of the recovery

% and goodness of fit of the curve.

%

% Written in MATLAB R2014a

%

% Written By Si Seet Chan, March 2015

% v5+ Revisions by Dave Mason [dnmason@liv.ac.uk]

% Centre for Cell Imaging, Liverpool University, UK

%

%-- Clear the workspace and command history

clear all

close all

clc

%-- Ask if there's a master file

useMaster=questdlg('Do you have a master file? Select No to make a new one or Cancel not to bother','Master');

if strcmp(useMaster,'Yes')

[mastFile,mastPath]=uigetfile('*','Select Master File');

%-- Load in existing data

[~, ~, mastData]=xlsread([mastPath mastFile]);

elseif strcmp(useMaster,'No')

mastPath=uigetdir('Select a folder in which to make a new Master File');

mastFile='masterFRAP.xls';

%-- No file, so store a new header

mastData={'Timestamp','Path','Filename','Plateau','Rate','R squared'};

end

%-- Get data from the workspace in the form of Time, Bleach (ROI1), Control (ROI2) and Background (ROI3)

%-- Find data in different formats optionally selcting multiple files

[inFile, inPath]=uigetfile({'*.*';'*.xls';'*.txt';'*.csv';'*.xlsx'},'select file(s)','multiselect','on');

if inPath<0;

disp('SCRIPT ERROR - Error in finding file')

return

end

%-- For multiselection of files to occur, need code to see whether is it

%-- multiple files or single files. Multiple files = char, single file = cell

if iscell(inFile)

for n=1:length(inFile)

%-- Get extension then test. If Excel, only import first sheet

[tempPath tempFile tempExt]=fileparts([inPath inFile{n}]);

if strfind(tempExt,'xls')>0

D=xlsread(fullfile([inPath inFile{n}]),1);

Data=D(:,1:4);

else

D=importdata(fullfile([inPath inFile{n}]));

Data=D.data;

end

Time=Data(:,1);

ROI1=Data(:,2);

ROI2=Data(:,3);

ROI3=Data(:,4);

%-- csv, old xls and txt format is 3, xlsx format is 4 (the code count the

%-- number to delete to replace the format

h=length(inFile{n})-strfind(inFile{n},'.');

%-- Find the number of PREBLEACH values

for i=1:size(ROI1,1)-1

NUM_PREBLEACH=i;

if (ROI1(i)-ROI1(i+1))/ROI1(i)>0.3

break

end

if NUM_PREBLEACH>round((0.5*(size(ROI1))))

disp('SCRIPT ERROR - Error in finding prebleach value')

return

end

end

% Find the mean of Background

Mean_ROI3=mean(ROI3);

% Background Substraction for ROI1 and ROI2

BGSUB_ROI1=ROI1-Mean_ROI3;

BGSUB_ROI2=ROI2-Mean_ROI3;

% Pre-bleach values for both ROI1 and ROI2

Pre_ROI1=ROI1(1:NUM_PREBLEACH);

Pre_ROI2=ROI2(1:NUM_PREBLEACH);

% Mean of Pre-bleach value for both ROI1 and ROI2

Mean_PreROI1=mean(Pre_ROI1);

Mean_PreROI2=mean(Pre_ROI2);

%Divide BG substraction for ROI1 and ROI2 with mean of Pre-bleach value

Norm_ROI1=BGSUB_ROI1/Mean_PreROI1;

Norm_ROI2=BGSUB_ROI2/Mean_PreROI2;

% Correct for non-specfic bleachng (Rt)

Rt=Norm_ROI1./Norm_ROI2;

% First Post Bleach Value (Rp)

Rp=Rt(NUM_PREBLEACH+1);

% Correct for Bleach Function

Bleach_Fraction=(Rt-Rp)/(1-Rp);

% Correct time for value after bleaching

Norm_Time=Time(NUM_PREBLEACH+1:end)-Time(NUM_PREBLEACH+1);

% Get the Norm recovery data from first post bleach value till end

Norm_Recovery=Bleach_Fraction(NUM_PREBLEACH+1:end);

% Curve Fitting

Equation='(a-b)*exp(-c*x)+b';

% where a= Value of Y where line cross Y axis

% b= Value of Y a infinity

% c= rate constant for graph

% Plotting of graph and set the starting point for the equation to fit the

% graph for normalised recovery

[fit1,fit1_gof,fit1_alg]=fit(Norm_Time,Norm_Recovery,Equation,'StartPoint',[1 1 0.01]);

% Flagged low Rsqaure value (less than 0.7 percent)

% plot residuals here against NormTime - don't forget to label axes and

% give the graph a title - use Figure to make a new figure fit1_alg.residuals

if fit1_gof.rsquare<0.7

figure('Color',[1 1 1]);

plot(Norm_Time,fit1_alg.residuals);

xlabel('Time (min)');

ylabel('Residuals');

title(['Residuals - ' inFile{n}(1:end-(h+1))], 'Interpreter','none');

legend('Residuals','location','northeast')

axis('square')

disp('SCRIPT ERROR - Low Rsquare value for graph')

F=getframe(gcf);

imwrite(F.cdata,[inPath inFile{n}(1:end-(h+1)) '_residuals.png'],'png');

end

% Flagged negative Rsquare value

if fit1_alg.exitflag<0

disp('SCRIPT ERROR - Fail to fit equation')

return

end

% Plotting of graphs (Raw Data and Normalised Recovery)

% Put both graphs into one plot

figure('Color',[1 1 1]);

% Set the figure position and size of the figure

set(gcf,'position',[100 100 960 430])

movegui(gcf,'center') %-- Do this to make sure that the figure isn't cut off (or including other windows) when exporting

% Plotting Raw Data - plot(Time,ROI1,Time,ROI2,Time,ROI3)

% Labeling of graph and axis, graphs are in square format so that it is

% easier to look at datapoints

subplot(1,2,1);

plot(Time,ROI1,Time,ROI2,Time,ROI3);

title(['FRAP Data - ' inFile{n}(1:end-(h+1))], 'Interpreter','none');

xlabel('Time (min)');

ylabel('Fluorescence Intensity (a.u.)');

legend ('Bleach','Control','Background','location','northeast')

xlim('auto')

ylim('auto')

axis('square')

% Plotting Normalised Recovery graph - plot(fit1,Norm_Time,Norm_Recovery)

% Labeling of graph, axis and setting limits to y axis

% Putting the graphs in square format so that it is easier to look at the

% datapoint

subplot(1,2,2);

plot(fit1,Norm_Time,Norm_Recovery);

title(['Normalised Recovery Graph - ' inFile{n}(1:end-(h+1))], 'Interpreter','none');

xlabel('Time (min)');

ylabel('Fractional Recovery');

legend('Normalised Recovery','Curve Fitting','location','southeast')

xlim('auto')

ylim([0 1.2])

axis('square')

% Save figure in png format

F=getframe(gcf);

imwrite(F.cdata,[inPath inFile{n}(1:end-h),'png']);

% Save workspace in mat format

save([inPath inFile{n}(1:end-h),'mat']);

%-- If selected, write out data to master

if strcmp(useMaster,'Yes') || strcmp(useMaster,'No')

c=fix(clock);

c=strcat(int2str(c(1)),'-',sprintf('%02d',c(2)),'-',sprintf('%02d',c(3)),'-',sprintf('%02d',c(4)),sprintf('%02d',c(5)));

%-- Append new data

mastData=vertcat(mastData,{c inPath inFile{n} num2str(fit1.b) num2str(fit1.c) num2str(fit1_gof.rsquare)});

%-- Write out master

xlswrite([mastPath '\' mastFile],mastData);

%-- Running batch to a master file, you probably don't want the images to stay open so...

close all

end

% Report data needed for analysis

disp(['----- ' inFile{n} ' -----'])

disp(['Y value at time infinity = ' num2str(fit1.b)])

disp(['Rate Constant =' num2str(fit1.c)])

disp(['R Square value for fitness of the equation=' num2str(fit1_gof.rsquare)])

end

%-- Single file selected

else

%-- Get extention then test. If Excel, only import first sheet

[tempPath tempFile tempExt]=fileparts([inPath inFile]);

if strfind(tempExt,'xls')>0

D=xlsread(fullfile([inPath inFile]),1);

Data=D(:,1:4);

else

D=importdata(fullfile([inPath inFile]));

% Data=D.data;

end

Time=Data(:,1);

ROI1=Data(:,2);

ROI2=Data(:,3);

ROI3=Data(:,4);

% csv, old xls and txt format is 3, xlsx format is 4 (make it into a

% variable so that the code could find out whether is it csv,xls,txt or

% xlsx format

h=length(inFile)-strfind(inFile,'.');

% Number of PREBLEACH values

for i=1:size(ROI1,1)-1

NUM_PREBLEACH=i;

if (ROI1(i)-ROI1(i+1))/ROI1(i)>0.3

break

end

if NUM_PREBLEACH>(0.5*(size(ROI1)))

disp('SCRIPT ERROR - Error in finding good prebleach value')

return

end

end

%Find the mean of Background

Mean_ROI3=mean(ROI3);

% Background Substraction for ROI1 and ROI2

BGSUB_ROI1=ROI1-Mean_ROI3;

BGSUB_ROI2=ROI2-Mean_ROI3;

% Pre-bleach values for both ROI1 and ROI2

Pre_ROI1=ROI1(1:NUM_PREBLEACH);

Pre_ROI2=ROI2(1:NUM_PREBLEACH);

% Mean of Pre-bleach value for both ROI1 and ROI2

Mean_PreROI1=mean(Pre_ROI1);

Mean_PreROI2=mean(Pre_ROI2);

%Divide BG substraction for ROI1 and ROI2 with mean of Pre-bleach value

Norm_ROI1=BGSUB_ROI1/Mean_PreROI1;

Norm_ROI2=BGSUB_ROI2/Mean_PreROI2;

% Correct for non-specfic bleachng (Rt)

Rt=Norm_ROI1./Norm_ROI2;

% First Post Bleach Value (Rp)

Rp=Rt(NUM_PREBLEACH+1);

% Correct for Bleach Function

Bleach_Fraction=(Rt-Rp)/(1-Rp);

% Correct time for value after bleaching

Norm_Time=Time(NUM_PREBLEACH+1:end)-Time(NUM_PREBLEACH+1);

% Get the Norm recovery data from first post bleach value till end

Norm_Recovery=Bleach_Fraction(NUM_PREBLEACH+1:end);

% Curve Fitting

Equation='(a-b)*exp(-c*x)+b';

% where a= Value of Y where line cross Y axis

% b= Value of Y a infinity

% c= rate constant for graph

% Plotting of graph and set the starting point for the equation to fit the

% graph for normalised recovery

[fit1,fit1_gof,fit1_alg]=fit(Norm_Time,Norm_Recovery,Equation,'StartPoint',[1 1 0.01]);

% Flagged low Rsqaure value (less than 0.7 percent)

% plot residuals here against NormTime - don't forget to label axes and

% give the graph a title - use Figure to make a new figure fit1_alg.residuals

if fit1_gof.rsquare<0.7

figure('Color',[1 1 1]);

plot(Norm_Time,fit1_alg.residuals);

xlabel('Time (min)');

ylabel('Residuals');

title(['Residuals - ' inFile{n}(1:end-(h+1))], 'Interpreter','none');

legend('Residuals','location','northeast')

axis('square')

set(gcf,'position',[100 100 450 450])

movegui(gcf,'center') %-- Do this to make sure that the figure isn't cut off (or including other windows) when exporting

disp('SCRIPT ERROR - Low Rsquare value for graph')

F=getframe(gcf);

imwrite(F.cdata,[inPath inFile(1:end-(h+1)) '_residuals.png'],'png');

end

% Flagged negative Rsquare value

if fit1_alg.exitflag<0

disp('SCRIPT ERROR - Fail to fit equation')

return

end

% Plotting of graphs (Raw Data and Normalised Recovery)

% Put both graphs into one plot

figure('Color',[1 1 1]);

% Set the figure position and size of the figure

set(gcf,'position',[100 100 960 430])

movegui(gcf,'center') %-- Do this to make sure that the figure isn't cut off (or including other windows) when exporting

% Plotting Raw Data - plot(Time,ROI1,Time,ROI2,Time,ROI3)

% Labeling of graph and axis, graphs are in square format so that it is

% easier to look at datapoints

subplot(1,2,1);

plot(Time,ROI1,Time,ROI2,Time,ROI3);

title(['FRAP Data - ' inFile(1:end-(h+1))], 'Interpreter','none');

xlabel('Time (min)');

ylabel('Fluorescence Intensity (a.u.)');

legend ('Bleach','Control','Background','location','northeast')

xlim('auto')

ylim('auto')

axis('square')

% Plotting Normalised Recovery graph - plot(fit1,Norm_Time,Norm_Recovery)

% Labeling of graph, axis and setting limits to y axis

% Putting the graphs in square format so that it is easier to look at the

% datapoint

subplot(1,2,2);

plot(fit1,Norm_Time,Norm_Recovery);

title(['Normalised Recovery Graph - ' inFile(1:end-(h+1))], 'Interpreter','none');

xlabel('Time (min)');

ylabel('Fractional Recovery');

legend('Normalised Recovery','Curve Fitting','location','southeast')

xlim('auto')

ylim([0 1.2])

axis('square')

% Save figure in png format

F=getframe(gcf);

imwrite(F.cdata,[inPath inFile(1:end-h),'png']);

% Save workspace in mat format

save([inPath inFile(1:end-h),'mat']);

%-- If selected, write out data to master

if strcmp(useMaster,'Yes') || strcmp(useMaster,'No')

c=fix(clock);

c=strcat(int2str(c(1)),'-',sprintf('%02d',c(2)),'-',sprintf('%02d',c(3)),'-',sprintf('%02d',c(4)),sprintf('%02d',c(5)));

%-- Append new data

mastData=vertcat(mastData,{c inPath inFile num2str(fit1.b) num2str(fit1.c) num2str(fit1_gof.rsquare)});

%-- Write out master

xlswrite([mastPath '\' mastFile],mastData);

end

% Report data needed for analysis

disp(['----- ' inFile ' -----'])

disp(['Y value at time infinity = ' num2str(fit1.b)])

disp(['Rate Constant =' num2str(fit1.c)])

disp(['R Square value for fitness of the equation=' num2str(fit1_gof.rsquare)])

end

%-- Clear the variable used to write out

clear mastData
